# Central venous access device management for children undergoing treatment for blood disorders and cancer: a descriptive international cross-sectional survey

**DOI:** 10.1007/s00520-025-09240-z

**Published:** 2025-02-12

**Authors:** Areum Hyun, Mari D. Takashima, Sabrina de Souza, Victoria Gibson, Thiago Lopes Silva, Patricia Kuerten Rocha, Siriporn Vetcho, Amanda J. Ullman

**Affiliations:** 1 School of Nursing, Midwifery and Social Work, Faculty of Health and Behavioural Sciences, The University of Queensland, St Lucia, QLD Australia; 2 School of Nursing and Midwifery, Griffith University, Nathan, QLD Australia; 3 Queensland Children’s Hospital, Children’s Health Queensland Hospital and Health Service, South Brisbane, QLD Australia; 4 Nursing Post-Graduation Program of the Universidade, Florianopolis, Federal de Santa Catarina Brazil; 5 Faculty of Nursing, Prince of Songkla University, Songkhla, Thailand

**Keywords:** Central vascular access device, Pediatric oncology, Catheter management, Global survey

## Abstract

**Purpose:**

This study aimed to explore current central vascular access device (CVAD) management practices in pediatric cancer care globally and compare practices across high-income and middle-income (i.e., upper middle-income and lower middle-income) countries.

**Methods:**

A descriptive cross-sectional international survey was conducted between May 2022 and December 2023. Clinicians involved in CVAD insertion/management for pediatric cancer patients were invited to participate through established clinical networks globally. The survey covered CVAD management practices, including dressings, securements, routine care, and maintenance procedures.

**Results:**

A total of 220 responses from 42 countries were received. Polyurethane dressings were most commonly used across all CVAD types (55–65/34.2–40.4). Bordered dressings were the primary securement method for all CVAD types (69–96/161, 42.9–59.6%). Routine practices varied, with 31/102 (30.4%) assessing CVADs hourly during infusion in high-income countries and 22 (37.3%) assessing every shift in middle-income countries. Heparin and normal saline were the commonly used solutions for flushing and locking. Chlorhexidine 2% in 70% alcohol was the predominant decontamination solution in high-income countries (64/102, 62.8%), while alcohol was more common in middle-income countries (22/59, 37.3%). Differences were observed between high-income and middle-income countries in the types of dressings, securements, and maintenance practices used.

**Conclusion:**

This study revealed significant variability in CVAD management practices for pediatric cancer care globally, particularly between high-income and middle-income countries. These findings highlight the need for standardized, evidence-based guidelines considering resource availability and contextual factors to improve CVAD management in pediatric oncology across diverse healthcare settings.

**Supplementary Information:**

The online version contains supplementary material available at 10.1007/s00520-025-09240-z.

## Introduction

Most children with malignant hematologic and oncological conditions require a central venous access device (CVAD) to administer chemotherapy, immunotherapy, and supportive therapies during their treatment [1]. While a CVAD is a pipe into the bloodstream that links a child to their treatment and supportive therapies, the underlying conditions and treatments of this population put them at a higher risk of adverse events associated with these devices. It is estimated that 1 in 3 CVADs in pediatric cancer care become infected (e.g., central line-associated bloodstream infections), thrombosed (e.g., venous thromboembolisms), or blocked during treatment [2]. These adverse events result in treatment disruptions and negatively impact their morbidity and mortality [3].

The complexity of these populations highlights the importance of providing specialized, high-quality care for CVAD management, and the practices must be aligned with high-quality evidence from studies involving this specific population. This is crucial because different types of CVADs—such as peripherally inserted central catheters (PICC) and totally implanted venous access devices (TIVAD)—vary in their materials (e.g., polyurethane, silicone), characteristics (e.g., lumens, valves), and intended dwell times (e.g., short-term to lifelong). Each type requires specific maintenance procedures and securement devices to prevent complications and ensure proper function [4–6]. For each of these care procedures, there are numerous products (e.g., polyurethane, silicone or gauze dressings) and approaches (e.g., hourly or less frequent site assessments) available for healthcare services, often without clear recommendations regarding their effectiveness for specific populations, device types, and available resources (e.g., human and equipment). This makes the selection of appropriate care practices challenging for clinicians [7].

While current routine practices with CVADs are often based on guidelines, many individual recommendations lack high-quality evidence [5, 8]. Regional variations in CVAD practice exist which may be due to tradition, opinion, and resource availability rather than best practice [9]. However, variation in clinical practice might fail to provide quality clinical outcomes. Recognizing the global variability in CVAD practice for pediatric cancer care serves as an initial step towards comprehending the underlying factors of such disparities (e.g., health equity), as it will allow for the development of targeted interventions tailored to each context. By identifying disparities and addressing specific needs, these interventions can be designed to be both effective and sustainable, ultimately improving outcomes for pediatric cancer patients worldwide [10]. Therefore, it is important to understand the current situation and identify the variations in usual practices to inform future management changes in specific contexts.

This study aimed to explore current CVAD management practices in pediatric cancer care. The objectives were to (1) describe CVAD management practices (dressing, securement, flushing, and locking practices), (2) compare them between devices, and (3) compare practices across economic classifications (high-income and middle-income countries).

## Methods

### Study design

A descriptive international cross-sectional survey was undertaken to explore CVAD-related practices in pediatric cancer care. Respondents were asked if CVADs were used in their practice for pediatric cancer care; if they responded “yes,” they moved on to the device section, which consisted of five CVAD-related practice domains: selection, insertion, management, removal, and complication management. In this study, the responses to the management domain were extracted and reported. The study was reported in accordance with the Consensus-based Checklist for Reporting of Survey Studies (CROSS) [11].

### Participants

Eligible participants were clinicians who manage CVADs in their pediatric cancer setting. The survey used a convenience snowball sampling method. The eligible participants were invited via established clinical trial networks and other associated networks. These included the Australian and New Zealand Children’s Haematology and Oncology Group (ANZCHOG), Cancer Nurses Society of Australia (CNSA), International Society of Paediatric Oncology (SIOP), and World Congress on Vascular Access (WoCoVA). Via an expression of interest pathway from these networks, a translated survey was also opportunistically distributed to Thailand and Brazil to collect inclusive and representative global responses on CVAD practices, particularly in countries where English is not the primary language. Due to the descriptive nature of the survey, there was no target sample size.

### Survey questionnaire

The survey questions were developed based on the Infusion Nursing Society (INS) Standards of Practice for Infusion Therapy [5], the American Society of Clinical Oncology (ASCO) [12], the Infectious Diseases Society of America (IDSA), American Society of Anesthesiologists [13], and the European Society for Medical Oncology Clinical Practice Guidelines [8].

The first part of the survey asked for demographic information about the participants and the characteristics of the healthcare organization the respondents represented. The CVAD-related practice management section consisted of dressing, securement, flushing, and locking practices. A group of pediatric cancer care interdisciplinary clinicians (*n* = 4) assessed the face validity and feasibility of the survey questionnaire [14]. The questionnaire was translated into Brazilian Portuguese and Thai by vascular access researchers who are native speakers of the language [15].

### Data collection

The online survey link was distributed to the participants between May 2022 to December 2023. The survey was integrated into the REDCap (Research Electronic Data CAPture; Vanderbilt, USA; http://project-redcap.org/). After obtaining permission to access potential participants from the clinical trials and clinical networks, a brief description, including the research purpose, was provided to potential participants. The team sent the online invitation with the survey link to the potential participants, via established networks. It took approximately 30 min –1 h to complete the survey. They were advised to contact the investigation team via email at any time with any questions. A reminder email was also sent 2 weeks later to the clinicians who did not participate in the online survey.

### Data analysis

Descriptive statistics, including frequency and percentages, were used to describe the data. Data was analyzed using the Stata Statistical Software: Release 15 (College Station, TX: StataCorp LP). Missing data are described throughout the results tables; however, due to the descriptive nature of the study, the missing values were not imputed. The heatmap diagram to visualize CVAD indications was developed using RAWGraphs [16]. Descriptive statistics, including frequency and percentages, were used to describe the data, and the data were also reported using the World Bank income level classification [17].

### Ethical consideration

Ethical approval was obtained from the University of Queensland (# 2023/HE000076). The potential participants were informed about their right not to participate in the survey, and they could stop at any point during the survey. Completion of the survey was considered to be consent. Confidentiality of survey information was maintained throughout the study. A web-based data management system, REDCap (Research Electronic Data Capture; Vanderbilt), was password protected and only accessible to designated personnel, and no identifiable information was collected.

## Results

### Characteristics of survey respondents

**Fig. 1:**
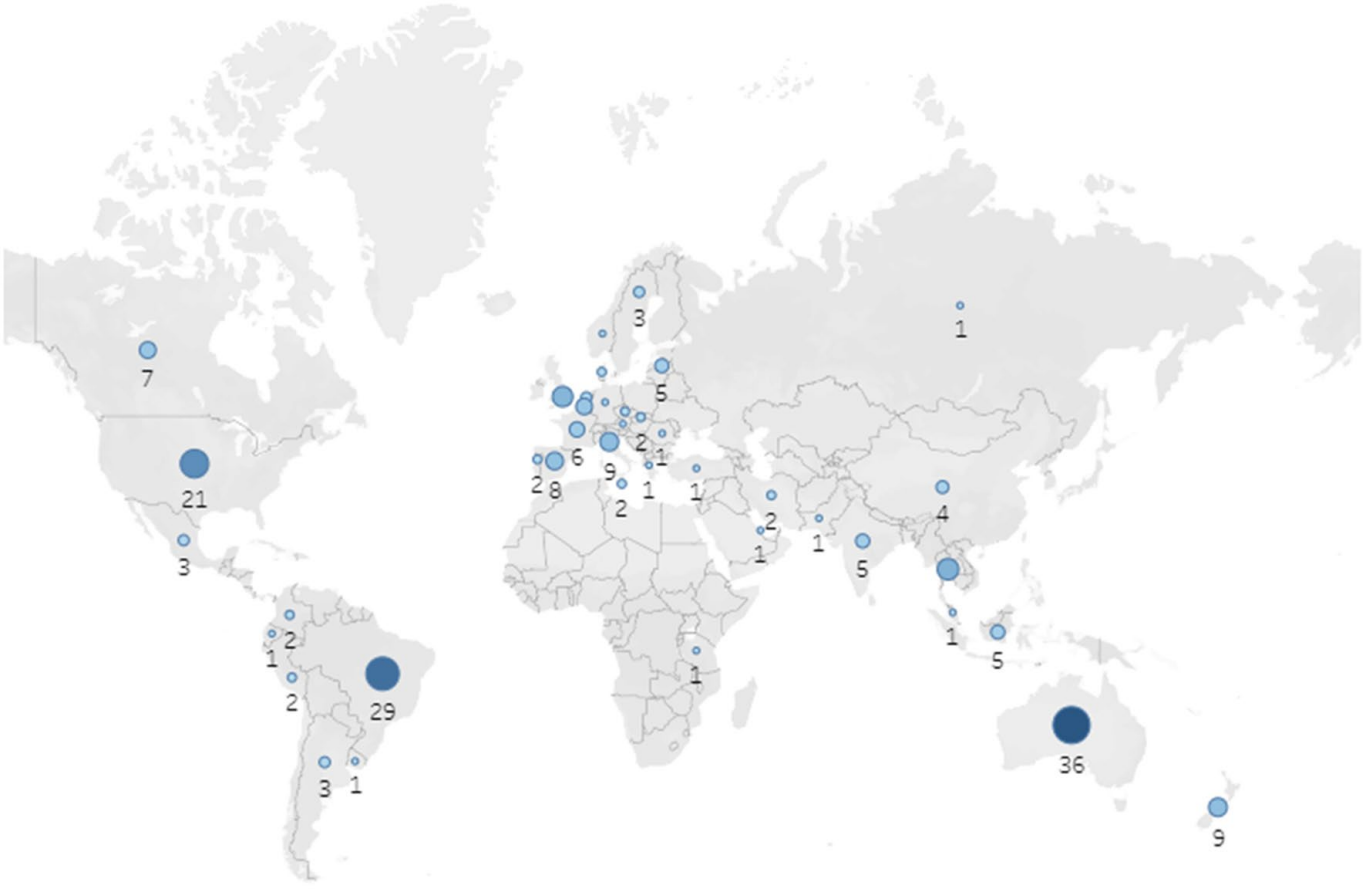
Map of survey respondents (*N* = 220)

A total of 220 participants from 42 countries responded to demographic information (see Fig. 1). Of these, 161 participants (73.2%) completed the device management section.

The demographic information is presented in Table 1. There were 144 respondents (65.5%) from high-income countries and 76 (34.6%) from middle -income countries. There was no response from low-income countries. Half the respondents were registered nurses (*n* = 84, 38.2%) and nurse practitioners (*n* = 30, 13.6%). Medical practitioners also responded (oncologists [*n* = 25, 11.4%] and anesthetists [*n* = 23, 10.5%]). The majority of high-income country respondents had more than 10 years of experience caring for patients with a cancer diagnosis (*n* = 104, 72.2%) whereas most middle-income country respondents had experience of fewer than 10 years (*n* = 43, 56.6%). Most of the respondents reported that they were practicing at third-level hospitals (*n* = 176, 80.0%) and public or government hospitals (*n* = 186, 84.6%).

**Table 1:** Demographics of survey respondents by group of countries classified by income level (*N* = 220)

Questions	Answers	Total	High-income countries	Middle-income countries
			*n* = 144	*n* = 76
		*n* (%)	*n* (%)	*n* (%)
Role of the respondents	Registered nurse	84 (38.2)	60 (41.7)	24 (31.6)
	Nurse practitioner	30 (13.6)	19 (13.2)	11 (14.5)
	Oncologist	25 (11.4)	11 (7.6)	14 (18.4)
	Anesthetist	23 (10.5)	19 (13.2)	4 (5.3)
	Surgeon	20 (9.1)	7 (4.9)	13 (17.1)
	Interventional radiologist	9 (4.1)	7 (4.9)	2 (2.6)
	Clinical nurse specialist	9 (4.1)	9 (6.3)	0 (0.0)
	Pediatrician	5 (2.3)	2 (1.4)	3 (4.0)
	Registrar in oncology/hematology/pediatrics	2 (0.9)	1 (0.7)	1 (1.3)
	Vascular access team	4 (1.8)	3 (2.1)	1 (1.3)
	Other (pharmacist, physiotherapist)	2 (0.9)	0 (0.0)	2 (2.6)
	Hematologist	2 (0.9)	2 (1.4)	0 (0.0)
	Intensivists	3 (1.4)	3 (2.1)	0 (0.0)
	No answer	2 (0.9)	1 (0.7)	1 (1.3)
Years of caring for patients with a cancer diagnosis	Less than 10 years	79 (35.5)	36 (25.0)	43 (56.6)
	10–19 years	64 (29.1)	47 (32.6)	17 (22.4)
	over 20 years	72 (32.7)	57 (39.6)	15 (19.7)
	Unknown	5 (2.3)	4 (2.8)	1 (1.3)
Level of hospital [18]	First level hospital	15 (6.8)	13 (9.0)	2 (2.6)
	Second level hospital	22 (10.0)	11 (7.6)	11 (14.5)
	Third level hospital	176 (80.0)	116 (80.6)	60 (79.0)
	Unknown	7 (3.2)	4 (2.8)	3 (4.0)
Types of hospitals*	Public or governmental	186 (84.6)	127 (88.2)	59 (77.6)
	Private or for-profit	29 (13.2)	13 (9.0)	16 (21.1)
	Unknown	7 (3.2)	4 (2.8)	3 (4.0)
Types of hospitals*	Pediatrics only	91 (41.4)	71 (49.3)	20 (26.3)
	Mixed	125 (56.8)	71 (49.3)	54 (71.1)
	Unsure	4 (1.8)	2 (1.4)	2 (2.6)
Annual number of children with cancer managed at the hospital	< 20	16 (7.3)	11 (7.6)	5 (6.6)
	20–49	20 (9.1)	9 (6.3)	11 (14.5)
	50–99	20 (9.1)	14 (9.7)	6 (7.9)
	100–299	65 (30.0)	48 (33.3)	17 (22.4)
	≥ 300	60 (27.3)	39 (27.1)	21 (27.6)
	Unsure	39 (17.7)	23 (16.0)	16 (21.1)

*Multiple options could be selected

### CVAD dressing and securement

**Fig. 2:**
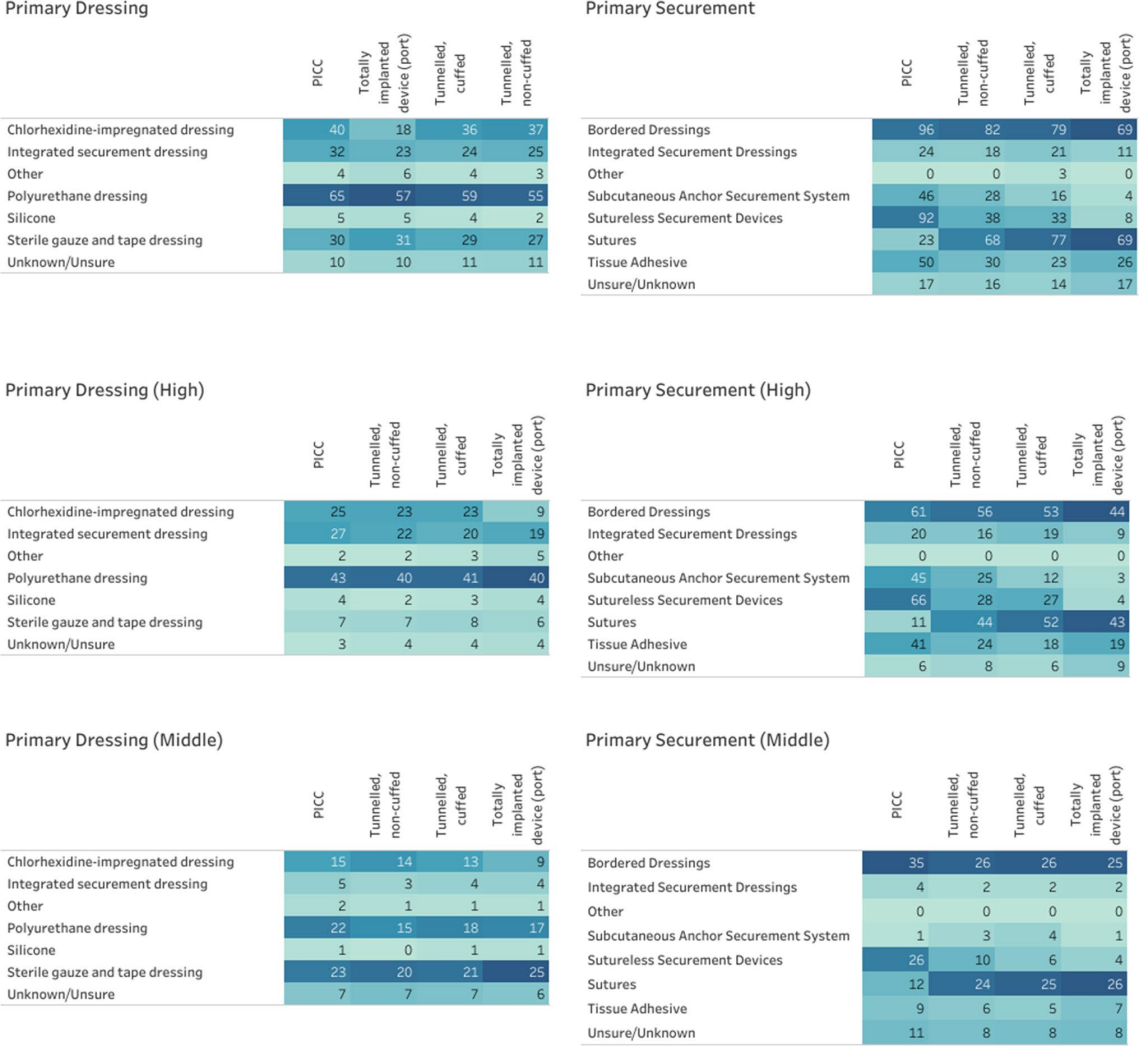
Dressings and securements by income level (total response *N* = 161, high-income *n* = 102; middle-income *n* = 59). Note: Darker colors represent a higher proportion. TIVAD primary securement for the port and the port needle

Transparent polyurethane dressings are the most commonly used dressing for all types of CVADs and are used in 40.4% (*n* = 65) of PICCs, 34.2% (*n* = 55) of tunneled non-cuffed CVADs, 36.7% (*n* = 59) of tunneled cuffed CVADs, and 35.4% (*n* = 57) of totally implanted venous access devices. The second most common dressing for PICCs, tunneled non-cuffed CVADs, and tunneled cuffed CVADs is chlorhexidine-impregnated dressings with 24.8% (*n* = 40), 23.0% (*n* = 37), and 22.4% (*n* = 36), respectively, while the second most common dressing for totally implanted venous access devices is sterile gauze and tape (*n* = 31, 19.3%). Polyurethane dressings with integrated securement are more common for all types of CVADs in high-income countries whereas sterile gauze and tape dressings and chlorhexidine-impregnated dressings are more commonly used in middle-income countries (see Fig. 2 and Supplementary Table 1).

Bordered dressings and sutures were the two most common primary CVAD securement approaches (Fig. 2). Bordered dressings are used in 59.6% (*n* = 96) of PICCs, 50.9% (*n* = 82) of tunneled non-cuffed CVADs, 49.1% (*n* = 79) of tunneled cuffed CVADs, and 42.9% (*n* = 69) of totally implanted devices. Sutures are used in 42.2% (*n* = 68) of tunneled non-cuffed CVADs, 47.8% (*n* = 77) of tunneled cuffed CVADs, and 42.9% (*n* = 69) of totally implanted devices, while sutureless securing devices are employed in 57.1% (*n* = 92) of PICCs. Clinicians from high-income countries reported use of more securement methods including integrated securement dressings, tissue adhesive and subcutaneous anchor securement systems for PICCs, tunneled non-cuffed CVADs, and tunneled cuffed CVADs (see Supplementary Fig. 1).

### Routine practices

Clinicians reported they conduct CVAD site assessment during an infusion every hour (*n* = 40, 24.8%) or every shift (*n* = 43, 26.7%); high-income countries assess every hour (*n* = 31, 30.4%), while those from middle-income countries assess every shift (*n* = 22, 37.3%; Table 2). Clinicians change CVAD dressings every 7 days (*n* = 132, 82%) and change totally implanted venous access device needles every 7 days (*n* = 117, 72.7%). Both income levels show similar practices in dressing changes and changing port (*totally implanted venous access device*) needles. Neutral fluid displacement needleless connectors were more commonly used (*n* = 62, 38.5%), followed by positive fluid displacement connectors (*n* = 41, 25.5%). Clinicians from middle-income countries used positive fluid displacement connectors more frequently (*n* = 17, 28.8%) than neutral fluid displacement connectors (*n* = 11, 18.6%). Chlorhexidine 2% in 70% alcohol is the common decontaminating solution for needleless connectors in high-income countries (*n* = 64, 62.8%), whereas alcohol is used in middle-income countries (*n* = 22, 37.3%).

**Table 2:** Routine practice by income level (total *N* = 161, high-income *n* = 102; middle-income *n* = 59). Abbreviations: *TIVAD*, totally implanted venous access device (port)

Questions	Answers	Total	High-income countries	Middle-income countries
		*n* (%)	*n* (%)	*n* (%)
Frequency of site assessment during infusion	Every hour	40 (24.8)	31 (30.4)	9 (15.3)
	Every 2–8 h	28 (17.4)	15 (14.7)	13 (22.0)
	Every shift	43 (26.7)	21 (20.6)	22 (37.3)
	Every 24 h	15 (9.3)	9 (15.3)	6 (5.9)
	Other	1 (0.6)	1 (1.0)	0 (0.0)
	Unsure/unknown	26 (16.2)	20 (19.6)	6 (10.2)
Frequency of dressing change during hospitalization	3 times weekly	15 (9.3)	5 (4.9)	10 (17.0)
	Every 7 days	132 (82.0)	93 (91.2)	39 (66.1)
	Greater than 7 days	5 (3.1)	1 (1.0)	4 (6.8)
	Other	4 (2.5)	1 (1.0)	3 (5.1)
Frequency of TIVAD needles change	Every 5 day	8 (5.0)	3 (2.9)	5 (8.5)
	Every 7 day	117 (72.7)	81 (79.4)	36 (61.0)
	Greater than 7 days	8 (5.0)	3 (2.9)	5 (8.5)
	Other	4 (2.5)	4 (3.9)	0 (0.0)
	Unknown/unsure	53 (32.9)	41 (40.2)	12 (20.3)
Needleless connectors	None	10 (6.2)	4 (3.9)	6 (10.2)
	Positive fluid displacement	41 (25.5)	24 (23.5)	17 (28.8)
	Neutral fluid displacement	62 (38.5)	51 (50.0)	11 (18.6)
	Negative fluid displacement	9 (5.6)	5 (4.9)	4 (6.8)
	Valved	1 (0.6)	1 (0.9)	0 (0.0)
	Unsure/unknown	37 (23.0)	18 (17.7)	19 (32.2)
Decontaminating solutions for needleless connectors	Chlorhexidine 2% in 70% alcohol	80 (49.7)	64 (62.8)	16 (27.1)
	Chlorohexidine 0.5% in 70% alcohol	25 (8.3)	12 (11.8)	13 (22.0)
	Alcohol only	45 (15.0)	23 (22.6)	22 (37.3)
	Others: BISEPTINE	1 (0.3)	1 (1.0)	0 (0.0)
	Unknow/unsure	47 (29.2)	37 (36.3)	10 (16.9)
	Chlorohexidine 0.5% in alcohol	1 (0.3)	0 (0.0)	1 (1.7)

### Flushing and locking practice during hospitalization

Clinicians reported using normal saline (*n* = 124, 77.0%) most frequently to flush CVADs and heparin (*n* = 95, 59.0%) to lock CVADs (Table 3). Both income levels show similar practices in dressing changes, flushing, and locking solutions.

**Table 3:** Flushing and locking practice by income level (total *N* = 161, high-income *n* = 102; middle-income *n* = 59)

Questions	Answers	Total	High-income countries	Middle-income countries
Flushing solutions during hospitalization	Heparin	56 (34.8)	34 (33.3)	22 (37.3)
	Normal saline	124 (77.0)	84 (82.4)	40 (67.8)
	Other: TauroLock	3 (1.0)	3 (2.9)	0 (0.0)
	Unknown/unsure	41 (25.4)	36 (35.3)	5 (8.5)
Locking solutions during hospitalization	Heparin	95 (59.0)	63 (61.8)	32 (54.2)
	Normal saline	77 (47.8)	52 (51.0)	25 (42.4)
	Chelating agents*	25 (15.5)	18 (17.7)	7 (11.9)
	Antibiotics	2 (1.2)	1 (1.0)	1 (1.7)
	Ethanol	4 (2.5)	2 (2.0)	2 (3.4)
	Other	1 (0.6)	1 (1.0)	0 (0.0)
	Unknown/unsure	43 (26.7)	37 (36.3)	6 (10.2)

*Example: Chelating agents (taurolidine citrate and tetrasodium-EDTA)

### Lock solutions after discharge

PICC, tunneled non-cuffed CVADs, and tunneled cuffed CVADs are routinely locked weekly when patients are receiving intermittent therapy or are outside the hospital (*n* = 84, 52.2%; *n* = 52, 32.3%; *n* = 72, 44.7%, respectively), whereas totally implanted devices are routinely locked every 4 to 6 weeks (*n* = 71, 43.5%). However, more than one-third of clinicians reported that they do not know how often CVADs are locked when patients are discharged (Table 4).

**Table 4:** Locking solutions when discharged from the hospital (*N* = 161 responses)

	Device types *n* (%)							
	PICC		TNC		TC		TIVAD	
	Total	High^a^	Total	High^a^	Total	High^a^	Total	High^a^
		Middle^b^		Middle^b^		Middle^b^		Middle^b^
12–24 h	4 (2.5)	4 (3.9)	1 (0.6)	1 (1.0)	0 (0.0)	0 (0.0)	1 (0.6)	1 (1.0)
		0 (0.0)		0 (0.0)		0 (0.0)		0 (0.0)
Weekly	84 (52.2)	53 (52.0)	52 (32.3)	35 (34.3)	72 (44.7)	53 (52.0)	16 (9.9)	10 (9.8)
		31 (52.5)		17 (28.8)		19 (32.2)		6 (10.2)
4 to < 6 weekly	11 (6.8)	4 (3.9)	14 (8.7)	7 (6.9)	24 (14.9)	9 (8.8)	70 (43.5)	47 (46.1)
		7 (11.9)		7 (11.9)		15 (25.4)		23 (39.0)
6 to < 8 weekly	2 (1.2)	1 (1.0)	3 (1.9)	1 (1.0)	3 (1.9)	2 (2.0)	13 (8.1)	8 (7.8)
		1 (1.7)		2 (3.4)		1 (1.7)		5 (8.5)
8 weeks +	1 (0.6)	0 (0.0)	0 (0.0)	0 (0.0)	1 (0.6)	0 (0.0)	16 (9.9)	11 (10.8)
		1 (1.7)		0 (0.0)		1 (1.7)		5 (8.5)
Never	1 (0.6)	1 (1.0)	1 (0.6)	1 (1.0)	0 (0.0)	0 (0.0)	3 (1.9)	3 (2.9)
		0 (0.0)		0 (0.0)		0 (0.0)		0 (0.0)
Other	6 (3.7)	4 (3.9)	3 (1.9)	2 (2.0)	3 (1.9)	3 (2.9)	1 (0.6)	1 (1.0)
		2 (3.4)		1 (1.7)		0 (0.0)		0 (0.0)
Unknown/unsure	58 (36.0)	44 (43.1)	55 (34.2)	44 (43.1)	53 (32.9)	42 (41.2)	55 (34.2)	43 (42.2)
		14 (23.7)		11 (18.6)		11 (18.6)		12 (20.3)

^a^High-income countries

^b^Middle-income countries

Abbreviations: *PICC*, peripherally inserted central catheter; *TNC*, tunneled; non-cuffed; *TC*, tunneled cuffed; *TIVAD*, totally implanted venous access device (port)

Percentage out of total responses

## Discussion

This international survey explored CVAD management practices in pediatric cancer care across 42 countries and compared them by high and middle-income countries. The study focused on key aspects of CVAD management, including dressing, securement, flushing, and locking practice. The survey highlighted differences in the use of various dressing materials, securement devices, and maintenance procedures, reflecting the complex landscape of CVAD management in pediatric oncology across different healthcare contexts.

Clinicians reported that the most frequently used dressing for CVADs is polyurethane dressing, followed by chlorhexidine-impregnated dressings. In middle-income countries, we found that sterile gauze and tape dressings are used more frequently than chlorhexidine-impregnated dressings. This pattern reflects the long-standing preference for polyurethane dressings, while chlorhexidine-impregnated dressings are also gaining popularity across various healthcare settings [2]. This study found that clinicians prefer regular polyurethane dressings for pediatric cancer patients. Although evidence suggests that chlorhexidine-impregnated dressings could reduce infections [7], it is important to note that the primary studies about chlorhexidine-impregnated dressings were mainly involved adult critical care, with limited generalizability to the pediatric cancer population [19]. The clinical guideline also suggests that no recommendation can be made for pediatric patients about the use of chlorhexidine-impregnated dressings to protect the site of CVADs due to the lack of evidence, and it is not recommended for premature neonates due to the risk of serious adverse skin reactions [5]. Therefore, further research is needed to develop a comprehensive approach, including procedures or algorithms, for managing CVAD sites in patients with a risk of impaired skin integrity beyond a single policy or guideline [5, 20].

In terms of securement devices for CVADs, boarded dressings are the most frequently used method across four different device types. Sutures are the second most common for tunneled non-cuffed CVADs, tunneled cuffed CVADs, and totally implanted venous access devices (ports), while sutureless securement devices are primarily used for PICCs. This aligns with current evidence suggesting sutureless securement devices performed better to secure PICCs [21]. Middle-income countries had a lower number of additional securement devices, such as tissue adhesive and subcutaneous anchor securement systems (SASS), while high-income countries reported using a variety of securement devices, suggesting that these devices may not be available in clinical practice in middle-income countries, despite evidence indicating that their use can increase CVAD dwell time and reduce complications [7, 22]. If there are limited resources for dressing and securement devices, it is necessary to conduct research and provide specific evidence in their context [23].

Routine practices, such as frequency of dressing change, totally implanted venous access device needle change, and flushing and locking solutions, are similar across different income levels, which indicates consistency or coherence to current evidence practice [5]. However, the frequency of CVAD site assessment during infusion was reported differently. Clinicians in high-income countries reported assessing CVAD sites hourly, whereas in middle-income countries, assessments were conducted every shift during infusion. This variation may reflect differences in staffing levels, resource availability, or local interpretations of best practices, highlighting the need for globally applicable yet contextually sensitive guidelines [24]. Both income-level countries reported normal saline as the most used for flushing and heparin as the most frequent solution for locking. The recent network meta-analysis found that chelating agents (such as taurolidine-citrate) and antibiotic locks were associated with lower odds of CVAD-associated bloodstream infections compared to heparinized saline [6]. However, this survey indicates that these advanced locking solutions are not widely adopted. When the patient is discharged, the CVAD is locked weekly for PICC, tunneled non-cuffed, and tunneled cuffed, and totally implanted venous access device (port) were only locked 4 to 6 weekly. The responses broadly align with CNSA guideline [25]. However, the guideline on lock frequency was primarily based on low-level evidence, including surveys, retrospective studies, small prospective studies, and expert opinion, highlighting the need for more robust research in this area. Concerningly, a third of responses reported unknown locking practices for long-term devices after discharge, indicating a significant knowledge gap in outpatient CVAD management.

Our findings emphasize the critical lack of evidence-based practice guidelines for CVAD management in pediatric oncology. This gap may be attributed not only to the limited number of studies, particularly randomized controlled trials (RCTs) [4, 5], but also to the lack of high-quality evidence specifically tailored to the pediatric cancer population and reflective of different global resource levels, which is necessary to generate context-specific evidence [7].

Future guidelines could incorporate tiered recommendations that provide options based on available resources, allowing healthcare providers to implement the best possible practices within their constraints. This approach has been successfully implemented in middle-areas of global health [26] and could benefit CVAD practices in pediatric oncology. By fostering research capacity and collaboration in resource-limited settings, we can develop more inclusive, applicable, and effective guidelines that address the unique challenges and opportunities in these contexts.

## Limitations

This study has several limitations that should be considered when interpreting its results. The use of convenience and snowball sampling methods may have introduced a sampling bias, potentially affecting the representativeness of global practices. As the survey relied on self-reported data, responses may be subject to recall bias or social desirability bias, which could influence the accuracy of the reported practices. Despite efforts to translate the survey into Brazilian Portuguese and Thai, language barriers may have limited participation from clinicians in non-English speaking countries, potentially underrepresenting certain global regions. Expanding language translations in future studies would help improve inclusivity and provide a more accurate representation of global perspectives. The categorization of countries into high-income and middle-income groups may oversimplify the complex economic landscapes and healthcare systems within these broad categories. Additionally, the survey provides a snapshot of practices at a specific time point and may not capture evolving practices or recent changes in CVAD management. Lastly, the potential for misinterpretation of technical terms or practices across different healthcare systems and cultures could have affected the consistency of responses. Given the challenges of collecting qualitative responses in this international survey, future research could incorporate open-ended questions to explore providers’ perspectives on improving CVAD management and care. These limitations highlight the need for a cautious interpretation of the findings and suggest areas for refinement in future global surveys on CVAD management practices.

## Conclusions

This international survey has highlighted variability in CVAD management practices in pediatric cancer care across different economic settings. Key differences in routine practices, dressing types, securement methods, and locking solutions reflect resource availability, local practices, and implementation challenges. Our findings highlight the need for developing resource-sensitive guidelines, increasing research efforts in resource-limited settings, enhancing education for outpatient CVAD management, and improving the dissemination of best practices globally.

## Supplementary Information

Below is the link to the electronic supplementary material.

Supplementary file1 (DOCX 421 KB)

## Data Availability

Due to privacy concerns, the datasets used and/or analyzed during the current study are available from the corresponding authors upon reasonable request.
