# Supplementary material for: Central venous access device management for children undergoing treatment for blood disorders and cancer: a descriptive international cross-sectional survey
Source: Support Care Cancer. 2025 Feb 12;33(3):179. doi: 10.1007/s00520-025-09240-z (PMC11821666; doi:10.1007/s00520-025-09240-z)
Supplement: Supplementary file 1 — Supplementary file1 (DOCX 421 KB) [file 520_2025_9240_MOESM1_ESM.docx]

**Supplementary Table 1**

**Primary dressings and securement (Total N=161 responses, High income n = 102, Middle income n = 59)**

|  | Device types n (%) | | | | | | | |
| --- | --- | --- | --- | --- | --- | --- | --- | --- |
|  | PICC | | TNC | | TC | | TIVAD | |
|  | Total | High | Total | High | Total | High | Total | High |
|  |  | Middle |  | Middle |  | Middle |  | Middle |
| Primary dressing | | | | | | | | |
| Polyurethane dressing | 65 (40.4) | 43 (42.2) | 55 (34.2) | 40 (39.2) | 59 (36.7) | 41 (40.2) | 57 (35.4) | 40 (39.2) |
|  |  | 22 (37.3) |  | 15 (25.4) |  | 18 (30.5) |  | 17 (28.8) |
| Sterile gauze and tape dressing | 30 (18.6) | 7 (6.9) | 27 (16.8) | 7 (6.9) | 29 (18.0) | 8 (7.8) | 31 (19.3) | 6 (5.9) |
|  |  | 23 (39.0) |  | 20 (33.9) |  | 21 (35.6) |  | 25 (42.4) |
| Chlorhexidine-impregnated dressing | 40 (24.8) | 25 (24.5) | 37 (23.0) | 23 (22.6) | 36 (22.4) | 23 (22.6) | 18 (11.2) | 9 (8.8) |
|  |  | 15 (25.4) |  | 14 (23.7) |  | 13 (22.0) |  | 9 (15.3) |
| Integrated securement dressing | 32 (19.9) | 27 (26.5) | 25 (15.5) | 22 (21.6) | 24 (14.9) | 20 (19.6) | 23 (14.3) | 19 (18.6) |
|  |  | 5 (8.5) |  | 3 (5.1) |  | 4 (6.8) |  | 4 (6.8) |
| Silicone | 5 (3.1) | 4 (3.9) | 2 (1.2) | 2 (2.0) | 4 (2.5) | 3 (2.9) | 5 (3.1) | 4 (3.9) |
|  |  | 1 (1.7) |  | 0 (0.0) |  | 1 (1.7) |  | 1 (1.7) |
| Other | 4 (2.5) | 2 (2.0) | 3 (1.9) | 2 (2.0) | 4 (2.5) | 3 (2.9) | 6 (3.7) | 5 (4.9) |
|  |  | 2 (3.4) |  | 1 (1.7) |  | 1 (1.7) |  | 1 (1.7) |
| Unknown/ Unsure | 10 (6.2) | 3 (2.9) | 11 (6.8) | 4 (3.9) | 11 (6.8) | 4 (3.9) | 10 (6.2) | 4 (3.9) |
|  |  | 7 (11.9) |  | 7 (11.9) |  | 7 (11.9) |  | 6 (10.2) |
|  | Device types n (%) | | | | | | | |
|  | PICC | | TNC | | TC | | TIVAD^c^ | |
|  | Total | High | Total | High | Total | High | Total | High |
|  |  | Middle |  | Middle |  | Middle |  | Middle |
| Primary securement | | | | | | | | |
| Sutures | 23 (14.3) | 11 (10.8) | 68 (42.2) | 44 (43.1) | 77 (47.8) | 52 (51.0) | 69 (42.9) | 43 (42.2) |
|  |  | 12 (20.3) |  | 24 (40.7) |  | 25 (42.4) |  | 26 (44.1) |
| Bordered dressings | 96 (59.6) | 61 (59.8) | 82 (50.9) | 56 (54.9) | 79 (49.1) | 53 (52.0) | 69 (42.9) | 44 (43.1) |
|  |  | 35 (59.3) |  | 26 (44.1) |  | 26 (44.1) |  | 25 (42.4) |
| Integrated securement dressing | 24 (14.9) | 20 (19.6) | 18 (11.2) | 16 (15.7) | 21 (13.0) | 19 (18.6) | 11 (6.8) | 9 (8.8) |
|  |  | 4 (6.8) |  | 2 (3.4) |  | 2 (3.4) |  | 2 (3.4) |
| Tissue adhesive | 50 (31.1) | 41 (40.2) | 30 (18.6) | 24 (23.5) | 23 (14.3) | 18 (17.7) | 26 (16.2) | 19 (18.6) |
|  |  | 9 (15.3) |  | 6 (10.2) |  | 5 (8.5) |  | 7 (11.9) |
| Sutureless securement device | 92 (57.1) | 66 (64.7) | 38 (23.6) | 28 (27.5) | 33 (20.5) | 27 (26.5) | 8 (5.0) | 4 (3.9) |
|  |  | 26 (44.1) |  | 10 (17.0) |  | 6 (10.2) |  | 4 (6.8) |
| Subcutaneous anchor securement system | 46 (28.6) | 45 (44.1) | 28 (17.4) | 25 (24.5) | 16 (9.9) | 12 (11.8) | 4 (2.5) | 3 (2.9) |
|  |  | 1 (1.7) |  | 3 (5.1) |  | 4 (6.8) |  | 1 (1.7) |
| Other | 0 (0.0) | 0 (0.0) | 0 (0.0) | 0 (0.0) | 3 (1.9) | 3 (2.9) | 0 (0.0) | 0 (0.0) |
|  |  | 0 (0.0) |  | 0 (0.0) |  | 0 (0.0) |  | 0 (0.0) |
| Unsure/Unknown | 17 (10.6) | 6 (5.9) | 16 (9.9) | 8 (7.8) | 14 (8.7) | 6 (5.9) | 17 (10.6) | 9 (8.8) |
|  |  | 11 (18.6) |  | 8 (13.6) |  | 8 (13.6) |  | 8 (13.6) |

*^a^ High-income countries*

^b^ *Other income countries*

*^c^ TIVAD securement for port and the port needle*

*Abbreviations: PICC: peripherally inserted central catheter, TNC: Tunneled, non-cuffed, TC: Tunneled cuffed, TIVAD: totally implanted venous access device (port)*

*Percentage out of total response (checked all that apply)*

Supplementary2 _ Survey questionnaire

# CLOCK – About your practice

**There are 7 questions about you and your hospital.**

Q1) What is your role? Oncologist

Haematologist Paediatrician


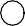

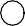

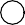

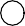


Registrar or currently undergoing specialty training in oncology, haematology and/or paediatrics

Nurse Practitioner Registered Nurse Other


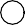

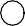

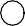


You selected 'Other' about your role. Please specify what your role is in your practice.

Q2) How long have you been caring for patients with a < 5 years cancer diagnosis? 5 - 9 years

10 - 19 years


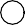

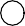

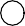

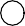


over 20 years

**About your hospital**

Q3) What country are you practicing in?

Q4) Where do you work [Hospital]?

First-level hospital: (e.g. Primary-level hospital, District hospital, Rural hospital, Community hospital, General hospital/ 50-250 beds)

Second-level hospital: (e.g. Regional hospital, Provincial Hospital, General hospital / 200-800 beds). Third level hospital: (e.g. National hospital, Central hospital, Academic, teaching or university hospital/ 300-1500 beds)


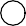
 First level hospital
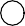
 Second level hospital
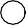
 Third level hospital

Q5) Types of Hospital
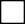
 Public or Governmental


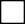
 Private or For-profit

Q6) Approximately how many children with cancer are
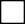
 < 20 managed in your hospital annually?
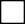
 20 - 49


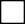
 50 - 99


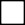
 100 - 299


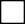
 ≥ 300


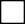
 Unsure

Q7) Is your hospital paediatrics only, or mixed adults and paediatrics?


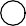
 Paediatrics only
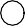
 Mixed

# CLOCK - Survey of Practice

There are a total of 32 questions regarding Central Venous Assess Devices (CVADs) in your practice.

**Indication (2 questions)**

**Q1) Select the common indications for each device in your practice (check all that apply).**

PICC Tunnelled,

non-cuffed CVAD

Tunnelled, cuffed

CVAD (e.g.,

permanent dialysis cath and Hickman cath)

Non-Tunnelled

CVAD (e.g.,

temporary dialysis cath- Vascath)

Totally implanted

device (e.g., port)

Duration of therapy less than 3 months

Duration of therapy exceeding 3 months

Intermittent / episodic therapy Continuous therapy

High complexity of infusion regimen (multiple infusates, multi lumen requirement)

Apheresis

Bone Marrow Transplant Solid tumours Haematological malignancy

Acute clinical instability of the patient (e.g., currently febrile)

Other

You selected 'Other' as an Indication of PICC. Please specify the common indication of PICC in your practice.

You selected 'Other' as an Indication of Tunnelled,

non-cuffed CVADs. Please specify the common indication of Tunnelled, non-cuffed CVADs in your practice.

You selected 'Other' as an Indication of Tunnelled, cuffed CVADs. Please specify the common indication of Tunnelled, cuffed CVADs in your practice.

You selected 'Other' as an Indication of Non-tunnelled CVADs. Please specify the common indication of

non-tunnelled CVADs in your practice.

You selected 'Other' as an Indication of ports. Please specify the common indication of ports in your practice.

Q2) Who is commonly involved in the decisions
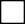
 Treating/primary clinician (e.g.,

surrounding device selection? oncologist/haematologist)


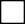
 Proceduralist (e.g., surgeon, anaesthetist, interventional radiologist)


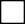
 Vascular access specialist (e.g., nurse practitioner)


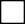
 Infectious disease specialist
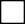
 Patient/family


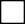
 Other (provide details)

You selected 'Other' as the above question about who is commonly involved in the decisions surrounding device selection. Please specify who is commonly involved in device selection.

**Selection (3 questions)**

**Q3) Do you routinely measure the catheter vessel ratio?**

PICC

Tunnelled, non-cuffed CVAD Tunnelled, cuffed CVAD Totally implanted device (port)

Yes No Unsure


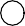

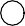

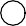

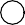

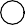

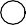

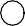

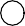

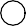


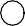

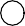

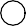


**Q4) What is the commonly chosen PICC/CVC material?**

Silicone

Plain polyurethane

Anti-thrombogenic polyurethane Antibiotic-coated polyurethane Anti-septic coated polyurethane Other

Unsure/Unknown

PICC Tunnelled, non-cuffed

CVAD

Tunnelled, cuffed

CVAD

Totally implanted

device (port)

You selected 'Other' for PICC material. Please specify the commonly chosen material of PICC.

You selected 'Other' for Tunnelled, non-cuffed CVAD material. Please specify the commonly chosen material

of Tunnelled, non-cuffed CVAD material.

You selected 'Other' for Tunnelled, cuffed CVAD material. Please specify the commonly chosen material

of Tunnelled cuffed CVADs.

You selected 'Other' for Port material. Please specify the commonly chosen material of Port in your practice.

Q5) What is the most popularly chosen PICC design?
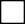
 External clamp


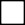
 Internal valve


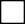
 Power injectable
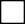
 Other


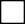
 Unsure/Unknown

You selected 'Other' for PICC design. Please specify

the most commonly chosen PICC design in your practice.

| **Placement (8 questions)**  **Q6) Who inserts CVAD at your site?** | | |
| --- | --- | --- |
| Anaesthetists  PICC | Surgeons | Interventional Nurses Other radiologists |
| Tunnelled, non-cuffed CVAD |  |  |
| Tunnelled, cuffed CVAD |  |  |
| Totally implanted device (port) |  |  |
| You selected 'Other' who inserts PICCs at your site. Please specify who inserts PICCs at your site. |  |  |
|  |  |  |
| You selected 'Other' who inserts Tunnelled, non-cuffed CVADs at your site. Please specify who inserts Tunnelled, non-cuffed at your site. |  |  |
|  |  |  |
| You selected 'Other' who inserts Tunnelled, cuffed CVADs at your site. Please specify who inserts Tunnelled, cuffed at your site. |  |  |
|  |  |  |
| You selected 'Other' who inserts ports at your site. Please specify who inserts ports at your site. |  |  |

**Q7) Where do you commonly insert the CVADs at your site?**

PICC

Tunnelled, non-cuffed CVAD Tunnelled, cuffed CVAD Totally implanted device (port)

Internal

jugular vein

Subclavian

vein

Femoral

vein

Basilic vein Brachial

vein

Cephalic

vein

Other

You selected 'Other' for the common insertion location of PICC. Please specify where you commonly insert the PICC in your practice,

You selected 'Other' for the common insertion location of Tunnelled non-cuffred CVADs. Please specify where

you commonly insert the Tunnelled non-cuffed CVADs in your practice,

You selected 'Other' for the common insertion location of Tunnelled, cuffed CVADs. Please specify where you

commonly insert the Tunnelled cuffed CVADs in your practice,

You selected 'Other' for the common insertion location of Port. Please specify where you commonly insert the

Port in your practice,

**Q8) What technology is used for CVAD insertion (including tip confirmation)?**

Ultrasound / Sonography

Intra-operative x-ray (including final tip position)

Post-insertion x-ray (after the patient has left the procedure room)

PICC Tunnelled, non-cuffed

CVAD

Tunnelled, cuffed

CVAD

Totally implanted

device (port)

ECG/EKG guidance Other Unsure/Unknown

You selected 'Other' for the technology used for PICC insertion. Please specify what technology you use for

PICC insertion in your practice,

You selected 'Other' for the technology used for tunnelled non-cuffed CVAD insertion. Please specify

what technology you use for tunnelled non-cuffed CVAD insertion in your practice,

You selected 'Other' for the technology used for tunnelled cuffed CVAD insertion. Please specify what

technology you use for tunnelled cuffed CVAD insertion in your practice,

You selected 'Other' for the technology used for port insertion. Please specify what technology you use for

port insertion in your practice,

Q9) Where is the goal CVAD tip location for upper
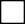
 Superior vena cava (upper third) extremity devices in your practice?
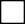
 Superior vena cava (mid-third)


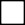
 Superior vena cava (lower third)
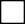
 Cavoatrial junction


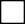
 Right atrium

Q10) If the CVAD is functioning (aspirating,
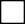
 Superior vena cava (upper third) injecting) which position would require the CVAD tip
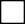
 Superior vena cava (mid-third) to be modified/exchanged/replaced?
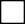
 Superior vena cava (lower third)


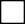
 Cavoatrial junction
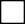
 Right atrium

Q11) If the CVAD has poor function(not aspirating)
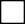
 Superior vena cava (upper third) which position would require the CVAD tip to be
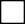
 Superior vena cava (mid-third)

modified/exchanged/replaced?
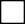
 Superior vena cava (lower third)
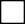
 Cavoatrial junction


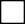
 Right atrium

**Q12) Which pain and sedation management commonly are administered to support CVAD insertion?**

Topical anaesthetic Intradermal lidocaine

Behavioural interventions (distraction, relaxation)

Procedural sedation General anaesthetic Other Unsure/unknown

PICC Tunnelled, non-cuffed

CVAD

Tunnelled, cuffed

CVAD

Totally implanted

device (port)

You selected 'Other' about PICC insertion pain management. Please specify what pain/sensation

management is commonly used to support PICC insertion.

You selected 'Other' about pain and sedation management during Tunnelled, non-cuffed CVAD

insertion. Please specify what pain/sensation management is commonly used to support Tunnelled,

non-cuffed CVAD insertion.

You selected 'Other' about pain and sedation management during Tunnelled, cuffed CVAD insertion.

Please specify what pain/sensation management is commonly used to support Tunnelled, cuffed CVAD

insertion.

You selected 'Other' about pain and sedation management during port insertion. Please specify what

pain/sensation management is commonly used to support port insertion.

**Q13) Which skin decontaminant is routinely used for CVAD insertion? (check all that apply)**

Chlorhexidine 2% in 70% alcohol

Chlorhexidine 0.5% in 70% alcohol

Povidone-iodine in alcohol Povidone-iodine without alcohol Other

Unsure/unknown

PICC Tunnelled, non-cuffed

CVAD

Tunnelled, cuffed

CVAD

Totally implanted

device (port)

You selected 'Other' about skin decontaminants during PICC insertion. Please specify what skin decontaminant is routinely used in your practice.

You selected 'Other' about skin decontaminants during Tunnelled, non-cuffed CVAD insertion. Please specify what skin decontaminant is routinely used in your practice.

You selected 'Other' about skin decontaminants during Tunnelled, cuffed CVAD insertion. Please specify what skin decontaminant is routinely used in your practice.

You selected 'Other' about skin decontaminants during port insertion. Please specify what skin decontaminant is routinely used in your practice.

**Management (10 questions)**

**Q14) What primary CVAD securements are used (check all that apply)**

Sutures

Bordered dressings (e.g.,Tegaderm Advanced®)

Integrated securement dressing (e.g., SorbaView® Shield)

Tissue adhesive

SSD (e.g.,StatLock®)

Subcutaneous anchor securement system (e.g.,securAcath)

PICC Tunnelled, non-cuffed

CVAD

Tunnelled, cuffed

CVAD

Totally implanted

device (port)

Other Unsure/Unknown

You selected 'Other' for a PICC securement method. Please specify what securements are used for PICC in your practice.

You selected 'Other' for a Tunnelled, non-cuffed CVAD securement method. Please specify what securements are

used for Tunnelled, non-cuffed CVADs in your practice.

You selected 'Other' for a tunnelled, cuffed CVAD securement method. Please specify what securements are

used for tunnelled, cuffed CVADs in your practice.

You selected 'Other' for a port securement method.

Please specify what securements are used for ports in

your practice.

**Q15) What are the primary CVAD dressings? (check all that apply)**

PICC

Tunnelled, non-cuffed CVAD Tunnelled, cuffed CVAD Totally implanted device (port)

Polyurethane

dressing

Sterile gauze

and tape dressing

Chlorhexidine-im

pregnated dressing

Integrated

securement dressing

Other (e.g.,

Silicone)

You selected 'Other' about a primary PICC dressing.

Please specify what are the primary PICC dressings in

your practice.

You selected 'Other' about a primary tunnelled,

non-cuffed CVAD dressing. Please specify what are the

primary tunnelled, non-cuffed CVAD dressings in your practice.

You selected 'Other' about a primary tunnelled, cuffed CVAD dressing. Please specify what the primary

tunnelled, cuffed CVAD dressings in your practice are.

You selected 'Other' about a primary port dressing. Please specify what the primary port dressings in your practice are.

Q16) How often is the CVAD site routinely assessed
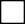
 Every hour

during the infusion?
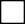
 Every 2 - 8 hours


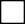
 Every shift


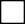
 Every 24 hours
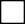
 Other


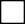
 Unsure/Unknown

You selected 'Other' for CVAD site skin assessment. Please specify how often the site assesses during infusion.

Q17) How often do you routinely change CVAD dressing
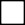
 3 times weekly when admitted to the hospital? (Check all that apply).
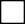
 Every 7 days


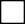
 Greater than 7 days
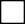
 Other

You selected 'Other' about dressing change. Please specify how often CVAD dressing changes in your practice.

Q18) What needless CVAD connectors are used? (check
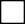
 None

all that apply)
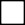
 Positive fluid displacement


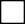
 Neutral fluid displacement
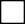
 Negative fluid displacement
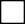
 Others


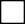
 Unsure/Unknown

You selected 'Other' about needless CVAD connectors. Please specify what needleless connectors are used in your practice.

Q19) Which decontaminating solutions are used for CVAD
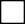
 Chlorhexidine 2% in 70% alcohol connectors?
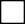
 Chlorohexidine 0.5% in 70% alcohol


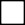
 Alcohol only
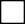
 Others

You selected 'Other' about decontaminating solution. Please specify what solutions are used for CVAD connectors in your practice.

Q20) What solution is routinely used for flushing
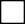
 Heparin

CVADs in your practice?
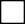
 Normal Saline


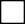
 Other

You selected 'Other' for CVAD flushing solutions. Please specify what solution is used for flushing CVADs in your practice.

**Q21) How often do you routinely lock CVADs when discharged from the hospital (i.e., seen in**

**outpatient)?**

Weekly

4 to < 6 weekly

6 to < 8 weekly

8 weeks Other

PICC Tunnelled, non-cuffed

CVAD


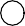

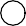

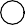

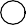

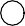

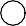

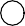

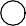

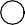

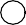


Tunnelled, cuffed

CVAD


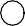

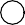

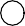

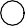

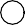


Totally implanted

device (port)

You selected 'Other' about locking period. Please specify how often you routinely lock PICC in your practice.

You selected 'Other' about locking period. Please specify how often you routinely lock Tunnelled, non-cuffed CVAD in your practice.

You selected 'Other' about locking period. Please specify how often you routinely lock Tunnelled, cuffed CVADs in your practice.

You selected 'Other' about locking period. Please specify how often you routinely lock totally implanted device (port) in your practice.

Q22) What solution is routinely used for locking the Heparin

CVADs? Normal Saline

Taurolidine & Citrate Antibiotics

Ethanol Other

You selected 'Other' about the CVAD locking solution. Please specify what solution is used for locking CVADs in your practice.

Q23) How often do you routinely change CVAD port Every 5 days

needles? (check all that apply) Every 7 days

Greater than 7 days Other

You selected 'Other' about port needle change. Please specify how often port needles are changed in your practice.

**Complication (9 questions)**

Q24) What is your first line of treatment for blocked Thrombolytic agents CVADs? Hydrochloric acid

Simple X-ray or equivalent

Fluoroscopy procedure (e.g., line-o-gram) Line exchange

Other

You selected 'Other' about the treatment of blocked CVADs. Please specify how you treat blocked CVADs in your practice.

Q25) What is your protocol for screening/diagnosis for Blood culture CABSI? (i.e. blood culture, tip culture, peripheral vs Tip culture

central DTP) Reservoir culture

Peripheral vs central DTP

Inspect VAD insertion site (e.g. erythema, edema, pain, tenderness, drainage)

Other

You selected 'Other' about a protocol for

catheter-associated bloodstream infection (CABSI). Please specify what your protocol for screening for CABSI is in your practice.

Q26) What is your treatment pathway when diagnosed with catheter-associated bloodstream infections (CABSI)?

Q27) What is the threshold of your commencement of antibiotic therapy due to suspected CABSI? (For example, when would you start treatment and what treatment is started).

Q28) Do you routinely screen/image for CVAD-associated always thrombosis for asymptomatic thrombosis? frequently

sometimes rarely

never

Q29) When a CVAD-associated VTE( Venous Remove the line as soon as possible thromboembolism) is diagnosed in an otherwise Anticoagulation prior to line removal asymptomatic patient, do you? Other

You selected 'Other'. Please specify what you do for

the treatment when a CVAD-associated VTE is diagnosed in an otherwise asymptomatic patient.

Q30) When a CVAD associated -VTE is diagnosed in a Remove the line as soon as possible blocked CVAD, do you? anticoagulated prior to line removal

Other

You selected 'Other'. Please specify what you do for

the treatment when a CVAD associated -VTE is diagnosed in a blocked CVAD.

Q31) What is your treatment pathway when diagnosed Unfractionated heparin

with CVAD-associated thrombosis? Lower Molecular Weight Heparin Dabigatran

Rivaroxaban Apixaban

Edoxabam Other

Unsure/Unknown

You selected 'Other' about the CVAD-associated thrombosis pathway. Please specify what is your treatment pathway after diagnosed with CVAD-associated thrombosis.

Q32) What are your most commonly used thrombolytic Urokinase agents? Reteplase

Tenecteplase Alfimeprase Alteplase

Other

You selected 'Other' for thrombolytic agents. Please specify what the commonly used thrombolytic agents in your practice are.
